# Quality of interaction between the nursing personnel and the informal caregivers of people with memory disorders: A systematic review and metasummary of qualitative studies

**DOI:** 10.1002/nop2.2029

**Published:** 2023-10-12

**Authors:** Johanna Rannikko, Jenny Paananen, Minna Stolt, Riitta Suhonen

**Affiliations:** ^1^ Department of Nursing Science University of Turku Turku Finland; ^2^ Satakunta Wellbeing Services County Pori Finland; ^3^ Turku University Hospital and Wellbeing Services County of Southwest Finland Turku Finland

**Keywords:** dementia, family members, interaction, memory disorders, nursing, professional communication, professional–family relations

## Abstract

**Aim:**

To explore the factors that affect the quality of interactions between nursing personnel and the informal caregivers of people with memory disorders.

**Design:**

Systematic review and metasummary of qualitative empirical research.

**Methods:**

The literature search targeted studies concerning the professional care interactions between nursing personnel and the informal caregivers of people with progressive memory disorders. The search in PubMed, CINAHL, PsycINFO and Scopus covered records from the earliest possible date up to December 2020. The data were summarised using a qualitative metasummary method. Preferred Reporting Items for Systematic reviews and Meta‐Analyses (PRISMA) checklist was used to validate the reporting process.

**Results:**

Ten articles were included. As presented in 33 statements, the factors affecting the quality of interactions were related to (1) expectations, (2) memory disorders, (3) interaction strategies, (4) time and place of interactions and (5) organisational aspects. Meeting the individual interactional needs of informal caregivers is recommended. The results provide guidance for improving the quality of interactions between nursing personnel and informal caregivers.

## 1 INTRODUCTION

Memory disorders are a worldwide problem causing major financial costs, human suffering, disability and dependency (Organisation for Economic Co‐operation and Development (OECD)/European Union, 2022; World Health Organization, 2017). The WHO has defined memory disorders as a public health priority and has stressed the importance of developing healthcare practices that serve people with memory disorders (PwMDs) and their informal caregivers, which includes increasing the competence of nursing personnel (World Health Organization, 2017).

Current views on healthcare support the concept of holistic care, which treats the care recipient as a whole and takes into account their physical, mental, emotional and social needs (Santana et al., 2018; World Health Organization, 2015). Person‐centred care is an approach that consciously strives to adopt the perspectives of individuals (World Health Organization, 2015) and to understand care recipients as co‐designers of care with respect to their own social nexuses (McCormack, 2004; Santana et al., 2018). According to recommendations, the informal caregivers of PwMDs should be recognised and involved in the care process for the benefit of the care recipients (World Health Organization, 2017).

The increasing diversity of the ageing population emphasises the relevance of collaboration with the care recipient's informal caregivers and of the social environment in professional care (Cruz‐Ortiz et al., 2011). From an interaction perspective, memory disorders pose particular demands for the involvement of informal caregivers. First, progressive memory disorders reduce individuals' ability to think, learn and remember, which interferes with their capacity to take care of themselves and to manage their daily life activities without help. In addition to weakening cognitive functions, memory disorders cause problems in language and communication, making it difficult for PwMDs to express their personal needs (Alzheimer's Disease International, 2021; World Health Organization, 2017). Therefore, the role of informal caregivers in a PwMD's everyday life is often substantial.

As informal caregivers are familiar with the care recipient's history, needs and preferences, they can provide valuable information and insight for professional care (Brannelly, 2011; Kiljunen et al., 2017). However, according to the World Health Organization (2017), providing care to PwMDs may affect informal caregivers' lives. They may experience helplessness, guilt, frustration, anger and a strong sense of duty (Alzheimer's Society, 2017). Compared with caregiving for people with other diseases, caregiving for PwMDs involves more stress and emotional burden (Cohen et al., 2014), which can make the interactions between nursing personnel and informal caregivers challenging (Park, 2010; Zaider et al., 2016). Given the progressive nature of memory disorders, older individuals become especially vulnerable and require advocates to speak up for them, thus making the involvement of informal caregivers highly important (Fetherstonhaugh et al., 2021; Kalaitzidis & Jewell, 2020). Studies have revealed the inappropriate treatment of PwMDs (Sormunen et al., 2007), a lack of interaction with and knowledge disseminated to their informal caregivers (Suhonen et al., 2015) and the need for effective communication (Puurveen et al., 2018) in the care settings of older people. Information on the factors affecting the quality of interactions between nursing personnel and informal caregivers regarding PwMDs' care is scarce. Therefore, the knowledge obtained from this study will help guide improvements in the interactions between nursing personnel and informal caregivers.

## 2 THE REVIEW

This study focuses on interactions, which refer to situations in which two or more people communicate or react to each other (Cambridge Dictionary, 2022). In a broad sense, interaction is a multidimensional concept that encompasses not only speaking, listening, observing and interpreting but also perceiving previous experiences, expectations and interpersonal relationships (Foa & Foa, 2012; King, 1981). This study investigates the interactions between nursing personnel and informal caregivers of PwMDs in a professional care context. Memory disorders in this context result from organic diseases of the brain that cause impairment of memory and affect other cognitive functions and behaviours. The care recipients had progressive memory disorders, but separate diagnoses or the stage of disease progression was not relevant for the purposes of this metasummary.

Interaction with informal caregivers is considered significant for nursing personnel to meet PwMDs' needs (Hamiduzzaman et al., 2020). Through interaction, nursing personnel and informal caregivers can explore each other's perceptions and preferences, enabling them to participate mutually in defining the goals of care and the means for achieving them (King, 1981). Effective interactions between nursing personnel and informal caregivers can enhance the nursing outcomes for PwMDs, reduce conflicts between nursing personnel and informal caregivers and improve the job satisfaction of nursing personnel (Robison et al., 2007). In addition, collaboration is known to reduce informal caregivers' caregiving stress and improve their satisfaction with care (Maas et al., 2004). Thus, besides ethical understanding, nursing personnel are required to have good interactional skills (Kiljunen et al., 2017) in order to support the care recipient's individuality, autonomy and quality of life (Moilanen et al., 2020). Effective interactions require mutual respect, willingness to understand each other, empathy and a safe atmosphere (Kiljunen et al., 2017).

Nevertheless, previous studies have shown that informal caregivers of PwMDs are not always satisfied with the amount of information they receive about care (Suhonen et al., 2015), and opportunities for interactions between nursing personnel and informal caregivers are often limited (Cohen et al., 2014; Puurveen et al., 2018). Nursing personnel find interacting with informal caregivers a challenging task (Utley‐Smith et al., 2009). Furthermore, although interaction skills are defined as a core competency for nursing personnel (Kiljunen et al., 2017), there is no clear consensus on the characteristics of high‐quality interaction with informal caregivers of PwMDs.

## 3 AIM

The aim of this systematic review and metasummary is to describe the factors affecting the quality of interactions between nursing personnel and PwMDs' informal caregivers in the professional healthcare context by summarising international qualitative evidence on the topic. The purpose is to promote the use of qualitative evidence in practice and to analyse the meaning of the findings in nursing practice and research. The information obtained will contribute to knowledge that will be used to promote interactions between nursing personnel and informal caregivers.

## 4 METHODS/METHODOLOGY

### 4.1 Design

This systematic review was conducted based on the qualitative metasummary method of Sandelowski and Barroso (2003), which is a quantitatively oriented approach to aggregation of qualitative findings (Sandelowski et al., 2007). A qualitative metasummary allows for integrating different research types to reach a comprehensive understanding of a phenomenon of interest by reporting and compiling the frequency and intensity of themes presented within qualitative studies (Sandelowski & Barroso, 2003). The purpose of using this method is not to produce a new analysis but to develop a structured description of the phenomenon under study, with an emphasis on the implications for practice and future research. The quantitative approach contributes to interpreting the state of research and to evaluating the validity of the findings. Using this method is in line with the aim of the current study, as the results may be used as bases for practical guidelines or further research (Sandelowski et al., 2007; Sandelowski & Barroso, 2003).

### 4.2 Search methods

The literature search targeted studies of professional care interactions between nursing personnel and the informal caregivers of people with progressive memory disorders. The search on the PubMed, CINAHL, PsycINFO and Scopus databases was conducted in December 2020.

The key search terms were ‘dementia’, ‘nurse’ or ‘nursing personnel’, ‘family member’ or ‘professional–family relations’, and the corresponding MeSH terms. As the word ‘interaction’ is abstract by nature and has several synonymous concepts, it was used as the main inclusion criterion, not a search term. ‘Dementia’, on the other hand, is an international and well‐established umbrella concept for a group of memory disorders. It was used as a search term with the corresponding MeSH term to cover different conditions caused by progressive memory disorders. In addition, ‘memory disorder’ and its alternative forms were used. Cognitive problems due to reasons other than progressive memory disorders were defined as grounds for exclusion. All search terms, the database‐specific search queries and the number of search results are presented in Table 1.

**TABLE 1:** Database search.

Database and date of search	Queries and limiters	Identified research articles	Selected by screening titles	Articles after removing duplicates	Selected by screening titles and abstracts	Selected by screening full texts
PubMed (new) 14.12.2020	((‘Dementia’[Mesh] OR ‘memory disorder*’ OR dementia* OR ‘cognitive problem*’ OR ‘cognitive disorder*’ OR ‘memory‐related disease*’ OR ‘degenerative brain disease*’) AND (‘Nurses’[Mesh] OR ‘nursing personnel’ OR nurse*) AND (‘family member*’ OR ‘informal caregiver*’ OR ‘family caregiver*’ OR ‘carer’ OR ‘carers’ OR ‘relative*’ OR ‘next of kin’ OR ‘significant other*’ OR ‘Professional‐Family Relations’[Mesh] OR ‘nurse‐caregiver’ OR ‘family‐staff’ OR ‘staff‐caregiver*’ OR ‘nurse‐carer’ OR ‘nurse‐family’) AND (English[lang]))	835	89			
Cinahl (EBSCO) 14.12.2020	((MH ‘Dementia+’) OR ‘memory disorder*’ OR dementia* OR ‘cognitive problem*’ OR ‘cognitive disorder*’ OR ‘memory‐related disease*’ OR ‘degenerative brain disease*’) AND ((MH ‘Nurses+’) OR ‘nursing personnel’ OR nurse*) AND ((MH ‘Professional‐Family Relations’) OR ‘family member*’ OR ‘informal caregiver*’ OR ‘family caregiver*’ OR ‘carer’ OR ‘carers’ OR ‘relative*’ OR ‘next of kin’ OR ‘significant other*’ OR ‘nurse‐caregiver’ OR ‘family‐staff’ OR ‘staff‐caregiver*’ OR ‘nurse‐carer’ OR ‘nurse‐family’) Limiters – Language: English Peer Reviewed	1066	113			
PsycINFO (EBSCO) 14.12.2020	(DE ‘Dementia’ OR DE ‘Neurodegenerative Diseases’ OR ‘memory disorder*’ OR dementia* OR ‘cognitive problem*’ OR ‘cognitive disorder*’ OR ‘memory‐related disease*’ OR ‘degenerative brain disease*’) AND (DE ‘Nurses’ OR DE ‘Nursing’ OR ‘nursing personnel’ OR nurse*) AND (DE ‘Family Members’ OR DE ‘Caregivers’ OR ‘family member*’ OR ‘informal caregiver*’ OR ‘family caregiver*’ OR ‘carer’ OR ‘carers’ OR ‘relative*’ OR ‘next of kin’ OR ‘significant other*’ OR ‘nurse‐caregiver’ OR ‘family‐staff’ OR ‘staff‐caregiver*’ OR ‘nurse‐carer’ OR ‘nurse‐family’) Limiters – Language: English Peer Review	641	53			
Scopus 14.12.2020	(TITLE‐ABS‐KEY((‘memory disorder*’ OR dementia* OR ‘cognitive problem*’ OR ‘cognitive disorder*’ OR ‘memory‐related disease*’ OR ‘degenerative brain disease*’))) AND (TITLE‐ABS‐KEY ((‘family member*’ OR ‘informal caregiver*’ OR ‘family caregiver*’ OR ‘carer’ OR‘ carers’ OR ‘relative*’ OR‘ next of kin’ OR ‘significant other*’ OR‘ nurse‐caregiver’ OR‘ family‐staff’ OR‘ staff‐caregiver*’ OR ‘nurse‐carer’ OR ‘nurse‐family’))) AND (TITLE‐ABS‐KEY ((‘nursing personnel’ OR nurse*))) AND (LIMIT‐TO (LANGUAGE,‘ English’))	876	119			
Total		3418	374	198	73	8

### 4.3 Inclusion/exclusion criteria

The inclusion criteria were as follows: (1) studies that examined the interactions between nursing personnel and the informal caregivers of PwMDs, (2) the interactions documented are related to the professional care of a person with a progressive memory disorder within any professional healthcare setting, (3) written in English and (4) empirical peer‐reviewed qualitative studies that met the criteria. The examined nursing personnel included registered nurses with nursing education and authorisation and other nursing personnel who are involved in the care of PwMDs. The professional titles and levels of education of other nursing personnel vary across countries. The informal caregivers of PwMDs may be relatives, extended family members, significant others or close friends, who form the care recipients’ personal social contexts (McCormack, 2004) and may be involved in the care process by directly providing support or care in daily life or arranging services for them (World Health Organization, 2017). As the focus is interaction, the setting of care or the aetiology of the progressive memory disorder is not relevant for the purpose of this review.

The following were the exclusion criteria: studies that (1) did not examine the interactions between nursing personnel and informal caregivers; (2) concerned one‐way communication, such as providing instructions, paper guides or information on websites; (3) focused on interactions that included other healthcare professionals, such as social workers or physicians; (4) investigated the interactions between nursing personnel and the care recipient or (5) examined the interactions between the care recipient and their informal caregivers. However, studies in which informal caregivers acted as informants in their interactions with healthcare professionals were generally included because it was not considered appropriate to assume that informal caregivers were aware of the various healthcare professions and the differences between them.

Furthermore, studies (6) in which nursing interventions were targeted at promoting the well‐being of the informal caregivers themselves, such as supporting them in coping as the primary caregivers; (7) in which the care recipients had cognitive problems due to reasons other than progressive memory disorders, such as trauma or congenital disabilities and (8) in which education was provided to nursing personnel or to informal caregivers about dementia as a disease were excluded. Because research on the topic is scarce, restrictions regarding the year of publication were not set in order to obtain as much information as possible. However, grey literature was excluded because finding all relevant citations would have been uncertain.

The examination covered all aspects of interactions that emerged from primary sources.

### 4.4 Search outcomes

**FIGURE 1:**
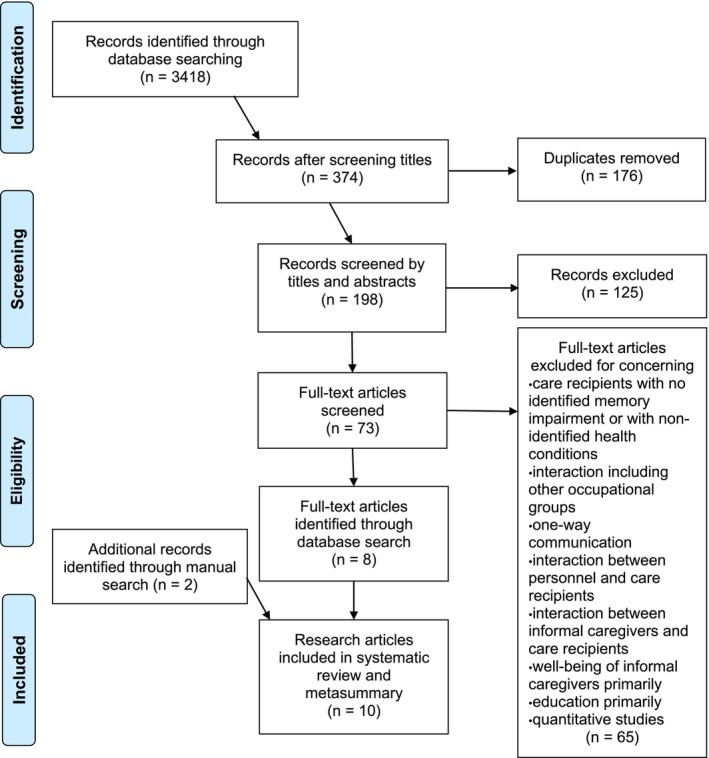
PRISMA 2020 flow diagram for identifying and selecting the studies included in the review. From: Page et al. (2021).

The database search produced 3418 citations and covered records from the earliest possible date up to December 2020. After a step‐by‐step process was implemented using the inclusion criteria, 374 citations were included based on their titles, 198 based on their titles and abstracts and 73 based on the full texts to reach an agreement between the two researchers (JR and RS) regarding the inclusion of eight full‐text articles. The search was complemented by a manual search of the reference lists of the retrieved articles, in which two more articles were included. In total, 10 articles were identified for the review and metasummary (Figure 1).

### 4.5 Quality appraisal

The research articles included were independently assessed for methodological rigour by two authors (JR and RS). They used the QualSyst criteria by Kmet et al. (2004), which are suitable for the quality appraisal of studies with different designs. The QualSyst checklist for assessing the quality of qualitative studies includes items central to assessing the internal validity of studies. Scores are given depending on the degree to which the criteria are met (yes/partial/no), and the total scores are reported against a maximum of 20 scores. That is, higher scores mean higher quality. The scoring system provides quantitative comparable information on the quality of research articles with different designs (Kmet et al., 2004).

### 4.6 Data abstraction and synthesis

All the content related to the interactions between nursing personnel and the informal caregivers of PwMDs was systematically labelled using NVivo12 data analysis software. The data were summarised using Sandelowski and Barroso's (2003) method. A qualitative metasummary allows for combining different types of research and presenting qualitative findings in an accessible form. Although the purpose of using this method is not to produce a new analysis but to develop a structured description of the phenomenon under study, the reviewers will have to define what will count as a finding, and how findings will be represented (Sandelowski & Barroso, 2003). The Preferred Reporting Items for Systematic reviews and Meta‐Analyses (PRISMA) checklist (Page et al., 2021) was used to guide reporting (File S1).

The qualitative metasummary method included three phases: (i) extraction of the relevant findings, (ii) abstraction of categorised sentences and (iii) calculation of effect sizes to verify the presence of findings in research on the topic. The purpose of calculating effect sizes is to ensure that the findings are neither over‐ nor under‐weighted and to explore the meanings of the findings in nursing research (Sandelowski et al., 2007; Sandelowski & Barroso, 2003).

In the first phase, the relevant sentences that were interpreted relative to this study were extracted independently from primary sources by one reviewer (JR). Some sentences were combined to retain their original meanings (*n* = 25). A total of 393 original sentences or combined sentences were identified and coded into categories according to their content. The other authors (JP, MS and RS) verified the extraction and categorisation. All the discrepancies were resolved by re‐reviewing the primary sources in detail.

**FIGURE 2:**
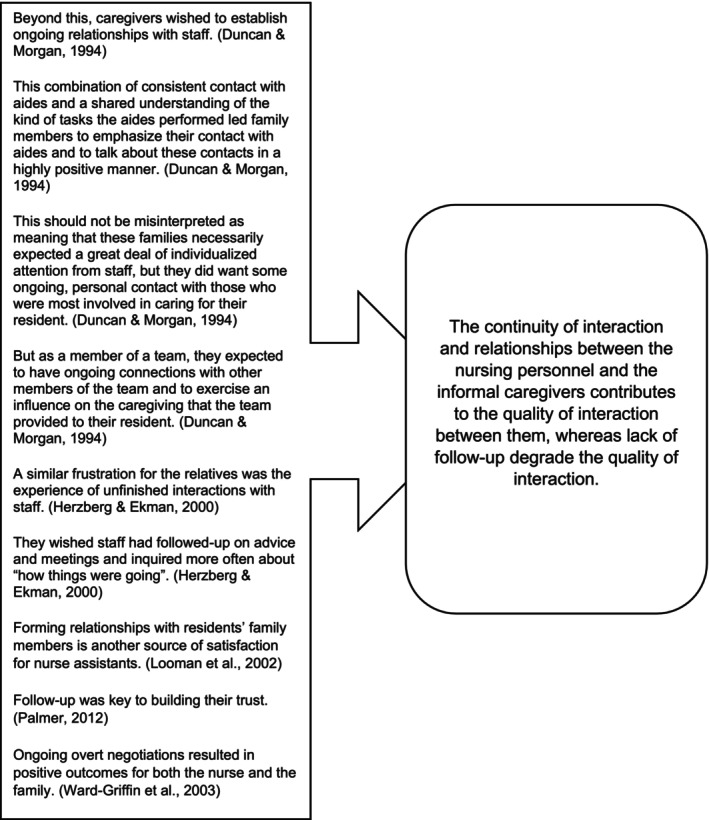
Example of abstraction of the original findings.

In the second phase, the sentences from each category were combined into abstracted statements by two reviewers JR and JP who worked together, and then they were verified by RS and MS. Some factors were described in primary research articles through either positive or negative aspects. These sentences were combined into the same statement. The abstracted statements were checked against each primary study to ensure that the original sentences appeared in them in either a positive or negative way, confirming the placement of the original sentences in the categories. Attempts were made to present the statements as informatively as possible. An example of the formation of an abstracted statement is presented in Figure 2.

In the third phase, the frequency effect sizes for the statements were calculated by taking the number of study reports containing the abstracted findings and dividing this number by the total number of reports. Finally, the intensity effect sizes were calculated by dividing the number of abstracted statements in the study by the total number of abstracted statements to determine the contributions of the primary studies to the corpus of statements.

## 5 RESULTS

### 5.1 Description of the studies included

The selected studies were diverse, having different approaches to the topic with different settings and following various methodologies or theoretical frameworks. The research articles were published between 1994 and 2016. Altogether, there were 596 participants in the studies. The sample sizes ranged from 11 to 114. The informants were informal caregivers in five studies and nursing personnel in two. Three studies included both types of informants, i.e., nurses and informal caregivers. Four of the studies were conducted in the US, two in Canada and one each in Sweden, the UK, Australia and Taiwan. Six studies took place in nursing homes, while four considered the care of home‐dwelling PwMDs. The data were collected through focus group interviews in two studies, in‐person interviews in five studies and a combination of these in two studies. The data of one study were collected by recording domiciliary meetings and in‐person interviews. The data analysis methods used varied, including thematic or comparative content analysis and discourse analysis. The perspectives on interactions were communication or the roles or relationships (Table 2).

**TABLE 2:** Selected research articles.

Author(s), Year of publication, Title (country)	Aim	Design, data collection, method participants	Main findings	QualSyst: Assessing the quality of qualitative studies^a^ (Kmet et al., 2004), with concerns
Adams (2001) The conversational and discursive construction of community psychiatric nursing for chronically confused people and their families (UK)	To get information of the work of community psychiatric nurses (CPNs) during domiciliary visits, particularly of their interaction with informal caregivers of people with diagnosed dementia	48 paired tape recordings at domiciliary meetings + 24 unstructured interviews of informal caregivers Conversation analysis and discourse analysis *n* = 48 (CPNs and relatives of home‐dwelling PwMD)	The three different communication formats that are locally and co‐operatively organised were identified. Through the (1) interview format, the nursing personnel elicits information about the informal caregiver and their confused care recipient; through the (2) information delivery format, control is exerted by the nursing personnel upon the informal caregiver and their care recipient; and through the (3) social interaction format, nursing personnel gains access to the private and hidden life of the family that would otherwise have been inaccessible and invisible. By using these conversational formats together with discursive practices, the work of the nursing personnel with the informal caregivers of PwMD may be accomplished	15/30 Strategic choice to recruit from caseloads of CPNs within five teams not described Verification procedures not evident No assessment about the personal impact of the researcher or the limitations
Duncan and Morgan (1994) Sharing the caring: family caregivers' views of their relationships with nursing home staff (USA)	To investigate the nature of the relationship between family caregivers and formal care staff, from the perspective of the family caregiver	30 recorded focus group interviews + 10 in‐person interviews Thematic content analysis *n* = 179 (informal caregivers of PwMD in nursing homes)	The relationships that the nursing personnel formed with the informal caregiver and the care recipient were crucial. The informal caregivers desired ongoing, personal contact with those who were most involved in caring for their care recipient. What mattered the most was the quality of care, which was not limited to technical tasks but also truly ‘caring about’	19/20 No evidence of using verification procedures at the phase of categorising the data No evidence of reflexivity of account
Fry et al. (2015) Emergency nurses perceptions of the role of family/carers in caring for cognitively impaired older persons in pain: A descriptive qualitative study (Australia)	To understand emergency nurses' perceptions of the role of family/carers in caring for the older cognitively impaired person experiencing pain	16 recorded focus group interviews Thematic content analysis *n* = 80 (emergency nurses from four emergency departments)	The role of the informal caregivers in building a clinical picture: valuable information source, which enhances the assessment process, the data and the care activities planned; deeper understanding, helping to communicate with the care recipient; sharing information is important The informal caregivers as hidden workforce: presence of the informal caregiver reduces agitation and confusion of the care recipients, the informal caregivers are sensitive to noticing changes in the care recipient's condition, which reduces the nursing personnel's workload and facilitates timely and suitable treatment for the care recipient The informal caregivers' roles in pain management decision‐making: could also interfere with the professional assessment of appropriate amount of analgesia, different care and pain management expectations between the nursing personnel and informal caregivers lead to tension Greater information sharing could reduce misunderstandings and expectation differences related to nursing practices	18/20 Sufficient sample size No assessment about the personal impact of the researchers
Heinrich et al. (2003) Seeking support: caregiver strategies for interacting with health personnel (Canada)	To explore the informal caregivers' perceptions of support from community resources while caring for a family member with dementia	62 in‐person interviews Secondary analysis, comparative content analysis on the foundation of symbolic interaction theory *n* = 20 (women, informal primary caregivers of home‐dwelling PwMD)	The informal caregivers' expectations of their caregiving role and their appraisal of the care recipient's cognitive abilities influenced their interactions with the nursing personnel Four interaction strategies: collaborating, getting along, ‘twigging’ (showing the needs of care‐recipient) and fighting/struggling Use of strategies varied according to degree of involvement in decision‐making and was accompanied by both negative and positive experiences of caregiving	16/20 No evidence of using verification procedures or reflexivity of the account
Hertzberg and Ekman (2000) ‘We, not them and us?’ Views on the relationships and interactions between staff and relatives of older people permanently living in nursing homes (Sweden)	To identify obstacles and suggest promoters concerning interaction between staff and relatives in connection with the care of people with dementia who are living in nursing home	18 recorded focus group interviews Observation, detailed notes Constant comparative method *n* = 24 (three staff members of nursing home, three relatives of PwMD, one professional group leader and one representative of Dementia Association and researcher as a silent non‐participant in each of the three groups)	The experiences that the nursing personnel and the informal caregivers had of each other were related to influence, participation, trust and ways to avoid conflicts. Experiences of situations were sometimes contradictory between the nursing personnel and informal caregivers. The results indicated that the potential for cooperation between the nursing personnel and informal caregivers in the care of the PwMD is not fully utilised	13/20 Context/setting partially explained Theoretical framework not described Wider body of knowledge partially described, but link to the study methods was not clear Sampling strategy not completely described No assessment about the personal impact of the researcher or the limitations
Alice Lau et al. (2008) Institutionalised elders with dementia: collaboration between family caregivers and nursing home staff in Taiwan (Taiwan)	To explore the process of development of collaborative relationship between family caregivers and nursing home staff	11 in‐person interviews, observing Constant comparative analysis Grounded theory *n* = 11 (family caregivers of PwMD in nursing homes)	‘Institutional social penetration’ was the most used dynamic process to form a collaborative relationship with the personnel The process included self‐disclosure, evaluations of care and integration strategies Within successful process, the informal caregivers were more likely to disclose information in more breadth and depth, to receive positive care evaluations, and to adopt multiple integration strategies	20/20 –
Looman et al. (2002) Impact of family members on nurse assistants: What helps, what hurts (USA)	To explore the nurse assistants' reports of both positive and negative behaviours exhibited by family members of PwMD	In‐person interviews Content analysis *n* = 114 (nurse assistants from 5 nursing homes)	Five categories of positive behaviours of informal caregivers: Expressions of thanks and appreciation to the nursing personnel The nursing personnel and the informal caregivers forming relationships with each other Showing trust in and respect for the nursing personnel Showing understanding about their care recipient's condition and nursing personnel's job burden Showing care for their care recipients in nursing home Four categories of negative behaviours of informal caregivers: Not trusting or respecting nursing personnel, or behaving inappropriately to them Not understanding the nursing personnel's job burden Complaining and blaming the nursing personnel or the nursing home Not showing appropriate care and concern for their cognitively impaired care‐recipient	18/20 Research questions or purpose not clearly described No assessment about the personal impact of the researchers
Mullins et al. (2016) Barriers to communication with a healthcare provider and health literacy about incontinence among informal caregivers of individuals with dementia (USA)	To examine barriers to communicating with healthcare professionals and health literacy about incontinence among different types of informal caregivers of individuals with Alzheimer Disease	Recorded focus groups, in‐person interviews, written surveys Descriptive secondary analysis, thematic content analysis *n* = 48 (informal caregivers of home‐dwelling PwMD)	Main themes of barriers emerged for each type of informal caregiver role: Emotive in nature for daughters: subject was intrusive into a personal part of their parent's life, private matter; the social stigma associated with incontinence. The daughters felt shame for their incontinent parents Experiential for both spouse caregivers: lack of knowledge and care capacity or preparedness System‐related for husbands: clinical routines as a barrier Relational for extended family/friends: being perceived as an outsider	18/20 Wider body of knowledge described but the link to the methods unclear Sampling method not fully described (reference to a parent study report)
Palmer (2012) Caregivers' desired patterns of Communication with Nursing Home Staff – Just TALKKK! (USA)	To uncover and disclose the patterns, gaps, concerns, dilemmas, conflicts, emotions and contradictions	In‐person interviews, field notes Interpretive study Hermeneutic phenomenology *n* = 15 (informal caregivers of PwMD at nursing home)	Six preferred patterns of communication, experienced by informal caregivers: tell, ask, know the care recipient in person, have knowledge about memory disorders, their progression, specific care and commonly used medications; and share the knowledge with the informal caregivers	18/20 Smaller sample size Sampling strategy not clearly described (reference to previous study) Reflexivity of the account partial
Ward‐Griffin et al. (2003) Relationships between families and registered nurses in long‐term‐care facilities: a critical analysis (Canada)	To critically examine the relationships between families and registered nurses caring for residents of a long‐term care facility for war veterans	34 in‐person interviews, tape‐recording, field notes, demographic data Progressive coding Critical ethnography *n* = 34 (17 family‐nurse dyads caring for PwMD in nursing homes)	Four types of personnel–informal caregiver relationships: conventional, competitive, collaborative, and carative The informal caregiver involvement in conventional and carative relationships was low, while the informal caregivers in competitive and collaborative relationships were highly involved in care Conventional and competitive relationships reflected a ‘resident‐focused’ approach to care, collaborative and carative relationships reflected a ‘family‐centred’ approach Negotiating strategies, and resulting consequences were described	19/20 No assessment about the personal impact of the researchers

^a^ The higher the score, the higher the quality of the research article.

### 5.2 Quality of the studies included

In general, the selected research articles were of good quality in terms of describing the research questions or objectives and using appropriate study designs. The contexts for the studies and their connections with the wider body of knowledge were described sufficiently in general. However, the underlying philosophies or the researchers' theoretical positions were often not identified, and there were some deficiencies in describing strategic choices in sampling. The data collection and analysis methods were clearly described and systematically presented in every study. The most common deficiency concerned the reflexivity of the accounts. In particular, the authors' likely personal influence on the studies was not assessed. In all of the selected studies, the conclusions were supported by the results. The quality appraisal is presented with scores and concerns in Table 2.

### 5.3 Metasummary of the findings

Summarising the data produced 33 abstracted statements describing the factors affecting the quality of interactions between nursing personnel and the informal caregivers of PwMDs. These statements were divided into five categories (A–E) based on substantive similarities. The categories were related to (A) expectations towards each other and towards nursing care, (B) factors associated with memory disorders, (C) interaction strategies, (D) time and place of interaction and (E) organisational factors. These categories are briefly described in the following text, but, in accordance with the metasummary method (Sandelowski & Barroso, 2003), the results are presented in Table 3 as abstracted statements at full length that are rich in content, together with the frequency effect sizes and references. In Table 3, the statements for each category are indicated in descending order of frequency effect size; i.e., the strongest statements come first. The frequency effect sizes of all statements ranged from 10% to 80%.

**TABLE 3:** Factors affecting the quality of interaction between the nursing personnel and informal caregivers.

Factors affecting the quality of interaction between the nursing personnel and the informal caregivers of people with memory disorders			
Improving (+)/degrading (−)^a^	Abstracted statements (*n* = 33)	Frequency effect size^b^ (%)	Research articles (*n* = 10)
(A) *Factors related to expectations towards each other or toward nursing care (n = 8)*			
+/−	(1) Conflicting expectations about each other's roles or goals and opportunities of nursing care are the main cause of conflict, frustration, misunderstanding, miscommunication, and dissatisfaction, even though good outcomes can be achieved from asymmetric roles as well, if both participants of the interaction accept their roles and power statuses and have similar expectations	80	Adams (2001), Alice Lau et al. (2008), Duncan and Morgan (1994), Fry et al. (2015), Heinrich et al. (2003), Hertzberg and Ekman (2000), Looman et al. (2002), and Ward‐Griffin et al. (2003)
+/−	(2) Recognising the informal caregivers and acknowledging their expertise about the care recipient promotes the quality of interaction between the nursing personnel and the informal caregivers, and oppositely, demonstrating indifference toward the informal caregivers degrades the quality of interaction	70	Alice Lau et al. (2008), Duncan and Morgan (1994), Heinrich et al. (2003), Hertzberg and Ekman (2000), Mullins et al. (2016), Palmer (2012), and Ward‐Griffin et al. (2003)
+/−	(3) The informal caregivers' positive evaluation of the quality of care improves the quality of interaction between the nursing personnel and the informal caregivers, and similarly, the negative evaluation of the quality of care degrades the quality of interaction	60	Alice Lau et al. (2008), Duncan and Morgan (1994), Heinrich et al. (2003), Hertzberg and Ekman (2000), Palmer (2012), and Ward‐Griffin et al. (2003)
+/−	(4) Encountering the care recipient as a person strongly indicates the good quality of care, and therefore affects the quality of interaction between the nursing personnel and the informal caregivers	50	Alice Lau et al. (2008), Duncan and Morgan (1994), Heinrich et al. (2003), Hertzberg and Ekman (2000), and Palmer (2012)
+	(5) Similarities in caregiving roles and experiences and common understanding of caregiving tasks between the informal caregivers and the nursing personnel facilitates the interaction; it is easier for the informal caregivers to approach the nursing personnel with lower education	50	Duncan and Morgan (1994), Heinrich et al. (2003), Hertzberg and Ekman (2000), Palmer (2012), and Ward‐Griffin et al. (2003)
+/−	(6) High level of trust and respect between the nursing personnel and the informal caregivers fosters the interaction and development of collaborative relationships and conversely, low level of trust and respect causes negative feelings and general dissatisfaction and degrades the quality of interaction	50	Adams (2001), Fry et al. (2015), Hertzberg and Ekman (2000), Looman et al. (2002), and Ward‐Griffin et al. (2003)
−	(7) The accentuated professional approach to care and interaction, or conflicting perceptions about power statuses of each other, degrades the quality of interaction between the nursing personnel and the informal caregivers since it may cause conflicts, passivate the informal caregivers and result in irritation and strain on both parties	30	Heinrich et al. (2003), Hertzberg and Ekman (2000), and Ward‐Griffin et al. (2003)
−	(8) Fear of negative consequences inhibits the interaction, since both the nursing personnel and the informal caregivers fear that transparency could lead to negative consequences for themselves or their relationship with the other participant of the interaction and, in addition, the informal caregivers fear negative consequences for the care recipient	30	Heinrich et al. (2003), Hertzberg and Ekman (2000), and Mullins et al. (2016)
(B) *Factors associated with memory disorders (n = 4)*			
−	(9) The informal caregivers of PWMD may suffer from emotional burden, which inhibits their interaction with the nursing personnel if their caregiving experience is accompanied with negative feelings (such as guilt, obligation, fatigue, loneliness, frustration, embarrassment, anxiety, or uncertainty) or with a sense of violating the care recipient's right to self‐determination or privacy, or if their relationship with the care recipient is problematic	80	Alice Lau et al. (2008), Duncan and Morgan (1994), Fry et al. (2015), Heinrich et al. (2003), Hertzberg and Ekman (2000), Looman et al. (2002), Mullins et al. (2016), and Ward‐Griffin et al. (2003)
+/−	(10) Memory disorders set expectations for the competence of the nursing personnel about the progression, treatment and handling of the behavioural symptoms and inadequate level of competence among the nursing personnel degrades the quality of interaction	60	Adams (2001), Duncan and Morgan (1994), Heinrich et al. (2003), Hertzberg and Ekman (2000), Mullins et al. (2016), and Ward‐Griffin et al. (2003)
NA	(11) Memory disorders produce special needs for sharing information between the nursing personnel and the informal caregivers, since the care recipient may not be able to provide the information about their current state or daily activities, baseline functions, medical and personal history or personal preferences, to guide the assessment of their needs, well‐being or the quality of care	40	Duncan and Morgan (1994), Fry et al. (2015), Heinrich et al. (2003), and Palmer (2012)
NA	(12) The appraisal of the care recipient's cognitive status affects the perceptions about the requisite level of informal caregiver participation	30	Fry et al. (2015), Heinrich et al. (2003), and Mullins et al. (2016)
(C) *Factors related to interaction strategies (n = 9)*			
+/−	(13) The amount of interaction affects the quality of the interaction since increasing the amount of open communication promotes the alignment of expectations and enables the best possible care, whereas lack of communication leads to making assumptions, errors of interpretation, impaired evaluations of care, frustration and negative behaviours between the nursing personnel and the informal caregivers	70	Alice Lau et al. (2008), Duncan and Morgan (1994), Fry et al. (2015), Heinrich et al. (2003), Hertzberg and Ekman (2000), Palmer (2012), and Ward‐Griffin et al. (2003)
+/−	(14) The breadth of the information provided promotes trust and quality of interaction between the nursing personnel and the informal caregivers, since the informal caregivers are willing to receive information about memory disorders and related symptoms, their progression and treatment as well as about the care recipient's daily activities, behaviours or changes in health status	60	Adams (2001), Alice Lau et al. (2008), Duncan and Morgan (1994), Hertzberg and Ekman (2000), Mullins et al. (2016), and Palmer (2012)
+/−	(15) The level of proactivity in opening discussion, asking questions and encouraging the informal caregivers to come forward with their questions and suggestions affects the quality of interaction between the nursing personnel and the informal caregivers as taking an initiative facilitates the development of collaborative relationships	60	Alice Lau et al. (2008), Fry et al. (2015), Heinrich et al. (2003), Hertzberg and Ekman (2000), Mullins et al. (2016), and Palmer (2012)
+/−	(16) The clarity of communication and confirming that the messages are correctly understood improves the quality of interaction between the nursing personnel and the informal caregivers, whereas unclear or indirect communication, language problems or other ambiguities degrade the quality of interaction	50	Adams (2001), Fry et al. (2015), Hertzberg and Ekman (2000), Mullins et al. (2016), and Palmer (2012)
+/−	(17) Mutuality in decision making affects the formation of collaborative and complementary relationships between the nursing personnel and the informal caregivers and helps in problem‐solving for the benefit of the care recipient, increases the informal caregiver's participation and confidence to care, and improves the nursing personnel's job satisfaction	50	Adams (2001), Alice Lau et al. (2008), Heinrich et al. (2003), Hertzberg and Ekman (2000), and Ward‐Griffin et al. (2003)
+/−	(18) Providing emotional support for the informal caregivers, such as affirming their work as a caregiver, providing time and information to adjust to their caregiving roles or showing compassion and understanding, improves the quality of interaction between the informal caregivers and the nursing personnel, but considering the informal caregiver as another patient in need of care might reduce the informal caregiver involvement and be stressful for the nursing personnel	40	Duncan and Morgan (1994), Fry et al. (2015), Heinrich et al. (2003), and Ward‐Griffin et al. (2003)
+	(19) Employing adaptive strategies, such as trying to look at the situation from the other's perspective, improves the quality of interaction between the nursing personnel and the informal caregivers	40	Alice Lau et al. (2008), Heinrich et al. (2003), Hertzberg and Ekman (2000), and Looman et al. (2002)
+	(20) Behaving informally, sharing some private information and adding personal dimensions into interactions could enhance the quality of interaction and construction of collaborative relationships between the nursing personnel and the informal caregivers	30	Adams (2001), Alice Lau et al. (2008), and Hertzberg and Ekman (2000)
+	(21) The quality of interaction between the nursing personnel and the informal caregivers improves if the nursing personnel consider that different types of informal caregivers may have different needs for the interaction with the nursing personnel; for example, children and spouses may feel differently about discussing intimate matters	30	Heinrich et al. (2003), Mullins et al. (2016), and Ward‐Griffin et al. (2003)
(D) *Factors related to time and place of interaction (n = 5)*			
+/−	(22) Transition phases, such as seeking for professional care or moving into a nursing home, are emotionally sensitive situations where sufficient interaction is relevant to the formation of relationships between the nursing personnel and the informal caregivers of PwMD, and the experiences from transition phases affect the quality of interaction even later	50	Alice Lau et al. (2008), Heinrich et al. (2003), Hertzberg and Ekman (2000), Palmer (2012), and Ward‐Griffin et al. (2003)
+/−	(23) The continuity of interaction and relationships between the nursing personnel and the informal caregivers contributes to the quality of interaction between them, whereas lack of follow‐up degrades the quality of interaction	50	Duncan and Morgan (1994), Hertzberg and Ekman (2000), Looman et al. (2002), Palmer (2012), and Ward‐Griffin et al. (2003)
+/−	(24) Explaining the planned actions beforehand improves the quality of interaction between the nursing personnel and the informal caregivers	20	Hertzberg and Ekman (2000) and Ward‐Griffin et al. (2003)
+/−	(25) The opportunity for discussions between the nursing personnel and the informal caregivers separately from the care recipients contributes to the quality of interaction	10	Mullins et al. (2016)
+	(26) Working together on practical tasks improves the quality of interaction between the nursing personnel and the informal caregivers	10	Hertzberg and Ekman (2000)
(E) *Organisational factors (n = 7)*			
+/−	(27) Not knowing who to talk to and having to talk to teams of the nursing personnel degrade the quality of interaction between the nursing personnel and the informal caregivers, and assigning a contact person could therefore improve the quality of interaction	40	Heinrich et al. (2003), Hertzberg and Ekman (2000), Palmer (2012), and Ward‐Griffin et al. (2003)
−	(28) Difficulties in accessing health care services or non‐functional contact practices degrade the quality of interaction between the nursing personnel and the informal caregivers	40	Heinrich et al. (2003), Hertzberg and Ekman (2000), Mullins et al. (2016), and Palmer (2012)
−	(29) Restricted organisational rules and task‐oriented division of professional roles do not support the implementation of personal care or equality of the informal caregivers, and this degrades the quality of interaction between the nursing personnel and the informal caregivers	30	Duncan and Morgan (1994), Palmer (2012), and Ward‐Griffin et al. (2003)
−	(30) The nursing personnel turnover weakens the flow of care information and opportunities for continuity of interaction	30	Duncan and Morgan (1994), Heinrich et al. (2003), and Hertzberg and Ekman (2000)
−	(31) Ambiguity, invisibility or absence of defined interaction and collaboration processes with the informal caregivers in the organisation degrades the quality of interaction between the nursing personnel and the informal caregivers	20	Fry et al. (2015) and Hertzberg and Ekman (2000)
−	(32) Insufficient flow of information among the nursing personnel and the lack of documentation systems that support the identification of life history and personal relationships degrades the quality of interaction with the informal caregivers	10	Hertzberg and Ekman (2000)
+/−	(33) A higher level of administrative and collegial support for the work and collaboration with the informal caregivers improves the quality of interaction between the nursing personnel and the informal caregivers, whereas lower level of support degrades the quality on interaction between them	10	Ward‐Griffin et al. (2003)

Abbreviation: NA, not applicable to record whether the factor improves or degrades the quality on interaction.

^a^ Marked with + if the factor was present in primary studies as improving the quality of interaction, with − if the factor was present as degrading the quality of interaction, and with +/− if both.

^b^ The number of research articles containing the abstracted finding divided by the total number of the research articles.

In general, 18 out of 33 factors or attributes appeared to have a two‐way impact on the quality of interactions, meaning that the existence or a higher level of an attribute improved the quality of interactions, whereas the absence or a lower level of an attribute degraded the quality of interactions. For example, informal caregivers' positive evaluations of the quality of care improve the quality of interactions between nursing personnel and informal caregivers, but negative evaluations degrade the quality of interactions. Some factors were only present in primary studies as either degrading or improving the quality of interactions, which applies particularly to organisational factors, in which shortcomings were mainly identified. For some factors, the positivity or negativity of factors could not be assessed. Information about whether an individual factor affects the interactions positively or negatively is presented in Table 3.

The statements in the first category concerned *expectations towards each other or towards nursing care* (A, *n* = 8), which affect the quality of interactions during encounters between nursing personnel and informal caregivers. The strongest individual statement in this category concerned the concordance or divergence of expectations. Conflicting expectations about each other's roles or goals and opportunities for nursing care cause conflict, frustration, misunderstanding and dissatisfaction but congruent expectations favourably affect the quality of interactions. In addition, nursing personnel could improve the quality of interactions by valuing informal caregivers and demonstrating personalised care of good quality. Having similarities in caregiving roles and being trustworthy and respectful also improve the quality of interactions. The accentuated professional approach and fear of negative consequences inhibit the interactions between nursing personnel and informal caregivers. In this category, there were both strongly indicated statements and more fragmented findings with lower frequency effect sizes. The frequency effect sizes ranged from 30% to 80%.

In the category of *factors associated with memory disorders* (B, *n* = 4), the strongest statement concerned the emotional burden of informal caregivers, which inhibited their interactions with nursing personnel. Memory disorders cause difficulties in assessing care recipient's needs or the quality of care because the care recipient might not be able to provide information to nursing personnel or to their informal caregivers. In addition, memory disorders affect perceptions of the requisite level of informal caregiver participation and the set expectations for the competence of nursing personnel. As informal caregivers desire information about the progression and treatment of the disease and about the handling of behavioural symptoms, an inadequate level of competence among nursing personnel degrades the quality of interactions. The factors associated with memory disorders had frequency effect sizes ranging from 30% to 80%.

*Factors related to interaction strategies* (C, *n* = 9) refer to the quantity, content and manner of interpersonal communication. The amount and extent of shared information and the level of proactivity in initiating discussions had the strongest indications within this category. The clarity of interaction, the level of mutuality in decision making and the strategies in providing emotional support were the factors that enhanced the quality of interactions, but in the absence of these, the interaction quality deteriorated. Being adaptive, behaving informally and considering the different interaction needs of different informal caregivers improved the quality of interactions with informal caregivers. The frequency effect size varied from 30% to 70%.

Within the category of *time and place of interaction* (D, *n* = 5), the strongest evidence concerned the relevance of sufficient interactions during the transition phase, such as moving into a nursing home, and the continuity of interactions in general. In addition, explaining the planned actions beforehand, providing the opportunity to discuss separately from the care recipient and working together on practical tasks could improve the quality of interactions between nursing personnel and informal caregivers. The frequency effect size ranged from 10% to 50%.

The fifth category consists of *organisational factors* (E, *n* = 7) related to the workforce or to organisational practices. The organisational statement with the largest frequency effect size of 40% concerned the impact of the absence or presence of a contact person and non‐functional contact practices. The task‐oriented division of professional roles, personnel turnovers, absence of defined collaboration processes and insufficient flow of information in the organisation degrade the quality of interactions between nursing personnel and informal caregivers. The level of administrative and collegial support also affects the quality of interactions. The statements on organisational factors were fragmented, as they had frequency effect sizes of 10%–40%.

The intensity effect sizes of the primary studies ranged from 15% to 79%. The three studies with the greatest intensity effect sizes of 52%–79% examined interactions related to professional care as a broad concept, as in the current study. The other studies examined a narrower area of interactions or only represented one point of view (of either nursing personnel or the informal caregivers). The intensity effect sizes of these studies ranged from 15% to 39% and are presented in Table 4.

**TABLE 4:** The intensity effect sizes of selected research articles.

Study by first author, year	Abstracted statements^a^	Intensity effect size^b^ (%)
Adams (2001)	1, 6, 10, 14, 15, 16, 20	21
Duncan and Morgan (1994)	1, 2, 3, 4, 5, 9, 10, 11, 13, 14, 18, 23, 29	39
Fry et al. (2015)	1, 6, 9, 11, 13, 15, 17, 18, 31	27
Heinrich et al. (2003)	1, 2, 3, 4, 5, 7, 8, 9, 10, 11, 12, 13, 16, 17, 18, 19, 21, 22, 27, 28, 30	64
Hertzberg and Ekman (2000)	1, 2, 3, 4, 5, 6, 7, 8, 9, 10, 13, 14, 15, 16, 17, 19, 20, 22, 23, 24, 26, 27, 28, 30, 31, 32	79
Alice Lau et al. (2008)	1, 2, 3, 4, 9, 13, 14, 16, 17, 19, 20, 22	36
Looman et al. (2002)	1, 6, 9, 19, 23	15
Mullins et al. (2016)	2, 8, 9, 10, 12, 14, 15, 17, 21, 25, 28	33
Palmer (2012)	2, 3, 4, 5, 11, 13, 14, 15, 17, 22, 23, 27, 28	39
Ward‐Griffin et al. (2003)	1, 2, 3, 5, 6, 7, 9, 10, 13, 16, 18, 21, 22, 23, 27, 29, 33	52

^a^ As numbered in Table 3.

^b^ Calculated by dividing the number of abstracted statements in the study by the total number of abstracted statements, rounded‐off percentages.

## 6 DISCUSSION

This systematic review and metasummary identified the factors affecting the quality of interactions between nursing personnel and the informal caregivers of PwMDs. They were related to (A) expectations towards each other and towards nursing care, (B) factors associated with memory disorders, (C) interaction strategies, (D) time and place of interaction and (E) organisational factors. These are discussed below, and their connections with the implementation of holistic care are considered, where appropriate.

The statements about expectations underline the importance of sharing information and clarifying the goals and opportunities of nursing care as prerequisites for a successful interaction. As expected, the strongest individual statement concerned the concordance or divergence of expectations regarding each other's roles or nursing care. Consistent with previous research (Natan, 2009), conflicting expectations are the root cause of non‐functional interactions between nursing personnel and informal caregivers. These results concerning expectations also confirmed the notion that the quality of care and interactions improves with a personalised approach (Fetherstonhaugh et al., 2021; Hamiduzzaman et al., 2020). The core of holistic care for PwMDs is to promote the continuation of the self and normality (Edvardsson et al., 2010), in which informal caregivers clearly contribute to care. However, it should be noted that informal caregivers' preferred levels of involvement are a complex issue influenced by factors such as their relationships with the care recipients or their own life situations (Puurveen et al., 2018), and that their preferred roles in relation to care are not immutable (Keefe & Fancey, 2000). The importance of clarifying expectations underlines the need to discuss wishes and expectations from the early stages of providing professional care.

The statements on memory disorders indicated that informal caregivers need information and support to deal with the symptoms of memory diseases and with their own emotions. Memory disorders produce special needs for sharing information. In the case of memory disorders in which the care recipients might be unable to express themselves and their preferences, the implementation of good‐quality care may depend on the biographical knowledge from their informal caregivers (see also Brannelly, 2011; Hamiduzzaman et al., 2020). Interactions with informal caregivers are crucial for nursing personnel to understand the care recipient's state of life and to meet their needs as a person. However, the strongest statement confirmed the notion that an emotional burden is strongly present in the care of PwMDs (Cohen et al., 2014), degrading the quality of interactions between nursing personnel and informal caregivers. As the relevance of informal caregiver participation is emphasised in this special group, the idea of providing support to the entire family throughout the care process (Cottrell et al., 2020; World Health Organization, 2020) could be useful. The ways of implementing this should be further explored (see Albinsson & Strang, 2003). As the data revealed that specific features are associated with interactions concerning the care of PwMDs, competence in questions related to memory disorders on the part of nursing personnel is required as a building block for successful interactions.

The factors related to interaction strategies provide practical guidance on encountering the informal caregivers of PwMDs and promoting the perseverance of the care recipients' own social nexuses (see Lao et al., 2019; McCormack, 2004). The primary sources revealed that informal caregivers' information needs are wide ranging. Information should be provided on the memory disorder and on the care recipient's day‐to‐day activities and behaviours, in addition to information about acute changes in health status. According to other studies, the interactions should also cover information about opportunities for the informal caregivers to participate in the care recipient's life in the professional care setting (Fetherstonhaugh et al., 2021; Thompson et al., 2020), which would further promote the implementation of individualistic care planning (Hamiduzzaman et al., 2020). The statements also suggested that it would be desirable to understand the individual interactional needs of different informal caregivers and to create individual solutions in order to encourage them to interact with nursing personnel.

The statements on the time and place of interactions indicated that the interactions between nursing personnel and informal caregivers should be given attention on an ongoing basis and not just, for instance, at the beginning of care. The fact that transition phases and the continuity of interactions had particular relevance to the quality of interactions (also Cottrell et al., 2020) underlines that efforts should be made to ensure the coherence of the care pathway and the cross‐organisational flow of information. Bélanger et al. (2017) found that either a positive or negative spiral is easily created, which is why investing in every contact is worthwhile. As many of the factors that related to interaction strategies or the time and place of interactions had a two‐way impact on the quality of interactions (i.e. the existence or a higher level of an attribute improved the quality of interactions, whereas the absence or a lower level of an attribute degraded it), intensifying nursing personnel's education on basic communication skills could be useful.

Organisational factors indicated that the adoption of the pursued holistic model of care that includes interactions with informal caregivers requires organisational development in various areas, including tools, personnel and practices. The findings on organisational factors were the most fragmented, but one might consider them the foundation that enables all of the above. For example, there is a lack of documentation system that supports the identification of care recipients' life histories and personal relationships. Hamiduzzaman et al. (2020) found that, rather than being individualistic, care plans are often fairly general, thus clearly making this an area for development. The statements concerning nursing personnel showed that the nursing personnel's persistence and competence affect the quality of interactions with informal caregivers, and these factors are also relevant for the implementation of sufficient, good‐quality care in general (see Henderson et al., 2017). The statements also pointed out that some simple practical improvements could enhance the quality of interactions between nursing personnel and informal caregivers. The latter should be provided with adequate information on when and how it is appropriate for them to contact nursing personnel and with whom care recipients' matters can be discussed. Obviously, a restrictive organisational culture and a rigid division of professional roles do not support interactions with informal caregivers or the implementation of individual care, which remains true in light of recent studies (Aaltonen et al., 2021; Bennett et al., 2017). The lack of defined collaboration practices was supported by Lindhardt et al. (2008), who found that the interactions between nursing personnel and informal caregivers more often occur by chance rather than through planned cooperation. Organisational cooperation strategies could help define the forms of cooperation, as well as on what issues, through which channels and when nursing personnel should systematically contact informal caregivers.

Altogether, the strongest statements underlined the importance of clarifying expectations, of recognising the value of informal caregivers as support for professional care and of increasing the overall amount of interactions in challenging situations in which the emotional burden associated with memory disorders may inhibit the interaction. The frequency effect sizes of the statements ranged widely (10%–80%). Statements with lower frequency effect sizes expand our understanding of the topic and indicate the need for further research in order to confirm such statements. Statements with stronger frequency effect sizes have already been clearly identified in research. Some of the factors were only present in primary studies as either degrading or improving the quality of interactions, but one could assume that they also worked in the other direction. This especially applies to organisational factors, in which shortcomings were mainly identified (also Puurveen et al., 2018). Many statements partly overlap, and individual statements are probably more or less related to one another; however, the relationship remains unclear in this summary. Given that many factors had two‐way effects on the quality of interactions, these statements show opportunities for improvement. As the statements contained much information derived using qualitative methods, they may provide practical guidance for the development of nursing practice, nurse education and organisational management.

### 6.1 Methodological considerations and limitations

The research team collaborated within each stage of the review process, and all decisions were made through discussion. The protocol was not pre‐registered or published. University library personnel were consulted in the creation of the search queries, and the literature search was targeted at four databases central to nursing science. No time limits were used, as the number of publications on the topic was known to be limited. Only English‐language, peer‐reviewed, qualitative empirical studies were selected, which helped increase the reproducibility of the review in the global clinical community. The use of a wider set of search terms, such as certain dementia diagnoses, could have produced more search results. However, the MeSH term tree for ‘dementia’ included all diagnosis‐based search terms and the search was complemented with ‘memory disorder’ and other corresponding terms. Not including grey literature meant that some relevant information may have been missed but a complementary manual search was conducted. The inclusion criteria applied to the care recipients and nursing personnel significantly limited the number of selected studies. Information was specifically sought about the specific features of the interactions associated with PwMDs in the context of nursing. The small sample may reduce accountability, but it shows the need for further research in this special context. The selected reports were mainly of good quality, but there were some concerns about some of them. The metasummary method seeks to include all valuable findings despite possible shortcomings in methodology or incomplete reporting (Sandelowski & Barroso, 2003), which supports the use of the method in relation to the practical purpose of the study. As the selected qualitative studies used various methodologies and approaches to the subject and as interactions are a challenging multidimensional concept covering a wide range of aspects (e.g. relationships, expectations, personal goals and interpretations; and spoken or non‐verbal messages), the quantitatively oriented aggregation of findings was a suitable approach for this research.

In the results interpretation, it should be noted that seven out of ten primary sources were more than a decade old, and practices may have evolved. However, the results of the earliest studies were in line with those of later ones. The dates of the studies do not diminish the value of the results when compared to today's nursing practices. Furthermore, the personal context of the researchers may have affected the extraction of findings, i.e., which findings were interpreted as relevant to the research question. However, alternative interpretations are also possible with respect to the abstraction of the original sentences. It should likewise be noted that care for PwMDs is organised differently across countries, so the interaction experiences may vary. Finally, the selected approach to the subject did not consider the care recipients' points of view or the impact of privacy policies, as confidentiality may contribute to limiting nursing personnel's interactions with informal caregivers.

## 7 CONCLUSION

The summarised findings underline the importance of clarifying expectations towards each other and towards nursing care, recognising the value of informal caregivers as support for professional care and increasing the amount of interactions in challenging situations in which the emotional burden associated with memory disorders and non‐functional organisational practices may degrade the quality of interactions between nursing personnel and informal caregivers. Functional organisational practices and supportive leadership are the cornerstone of improving the quality of interactions between nursing personnel and PwMDs' informal caregivers. Competence in memory disorders and communication skills are the building blocks, and continuous clarification of expectations towards each other and towards nursing care form the covering roof that promotes the quality of interactions between nursing personnel and informal caregivers.

### 7.1 Relevance to clinical practice

The results of this review provide guidance for improving the quality of interactions between nursing personnel and informal caregivers. First, the breadth, clarity and continuity of interactions with informal caregivers should be improved to avoid conflicts, to develop a satisfactory relationship between nursing personnel and informal caregivers and to provide the best possible care to PwMDs. It would be desirable to understand the different interactional needs of individual informal caregivers in order to encourage them to collaborate. Second, nursing personnel's interactional competence can be strengthened by developing their basic interaction skills and their knowledge about the progression, treatment and handling of the behavioural symptoms of memory disorders. Finally, both administrators and educators should pay special attention to ensuring that interactions with informal caregivers are identified as necessary support for nursing work that should be enabled and promoted in healthcare organisations. There is a need to develop practices and clear directions for collaboration with informal caregivers in a way that encourages the latter to interact with nursing personnel and that supports the individualisation of care for PwMDs.

## AUTHOR CONTRIBUTIONS

The conception and design of the study: JR, RS, MS. Acquisition of data: JR, RS. Analysis and interpretation of data: JR, JP, RS, MS. Drafting the article: JR, JP, RS. Revising it critically for important intellectual content: JR, JP, MS, RS.

## FUNDING INFORMATION

The systematic review and the report were conducted as part of the project Alongside Dementia funded by the Academy of Finland (grant number 331555). The study was also funded by the Turku University Hospital (special grant in aid VTR 13238). The study was not registered or prepared with a protocol and the funders did not influence conducting or reporting the review.

## CONFLICT OF INTEREST STATEMENT

The authors declare no conflicts of interest.

## ETHICS STATEMENT

This systematic review of previously published research articles did not require ethical approval or patient consent.

## Supporting information


File S1.

## Data Availability

Data sharing is not applicable to this article as no new data were created or analyzed in this study.
